# Approaches to transitioning women into and out of prevention of mother‐to‐child transmission of HIV services for continued ART: a systematic review

**DOI:** 10.1002/jia2.25633

**Published:** 2020-12-29

**Authors:** Tamsin K Phillips, Chloe A Teasdale, Amanda Geller, Bernadette Ng'eno, Pheposadi Mogoba, Surbhi Modi, Elaine J Abrams

**Affiliations:** ^1^ Division of Epidemiology and Biostatistics School of Public Health & Family Medicine University of Cape Town Cape Town South Africa; ^2^ Centre for Infectious Diseases Epidemiology & Research School of Public Health & Family Medicine University of Cape Town Cape Town South Africa; ^3^ ICAP‐Columbia University Mailman School of Public Health New York NY USA; ^4^ Department of Epidemiology Mailman School of Public Health New York NY USA; ^5^ Department of Epidemiology and Biostatistics CUNY Graduate School of Public Health & Health Policy New York NY USA; ^6^ US Centers for Disease Control and Prevention (CDC) Atlanta GA USA; ^7^ College Physicians and Surgeons Columbia University New York NY USA

**Keywords:** transition, transfer, prevention of mother‐to‐child transmission, linkage, continuity of care, antiretroviral therapy

## Abstract

**Introduction:**

Women living with HIV are required to transition into the prevention of mother‐to‐child transmission of HIV (PMTCT) services when they become pregnant and back to ART services after delivery. Transition can be a vulnerable time when many women are lost from HIV care yet there is little guidance on the optimal transition approaches to ensure continuity of care. We reviewed the available evidence on existing approaches to transitioning women into and out of PMTCT, outcomes following transition and factors influencing successful transition.

**Methods:**

We searched PubMed and SCOPUS, as well as abstracts from international HIV‐focused meetings, from January 2006 to July 2020. Studies were included that examined three points of transition: pregnant women already on ART into PMTCT (transition 1), pregnant women living with HIV not yet on ART into treatment services (transition 2) and postpartum women from PMTCT into general ART services after delivery (transition 3). Results were grouped and reported as descriptions of transition approach, comparison of outcomes following transition and factors influencing successful transition.

**Results & discussion:**

Out of 1809 abstracts located, 36 studies (39 papers) were included in this review. Three studies included transition 1, 26 transition 2 and 17 transition 3. Approaches to transition were described in 26 studies and could be grouped into the provision of information at the point of transition (n = 8), strengthened communication or linkage of data between services (n = 4), use of transition navigators (n = 12), and combination approaches (n = 4). Few studies were designed to directly assess transition and only nine compared outcomes between transition approaches, with substantial heterogeneity in study design, setting and outcomes. Four themes were identified in 25 studies reporting on factors influencing successful transition: fear, knowledge and preparedness, clinic characteristics and the transition requirements and process.

**Conclusions:**

This review highlights that, despite the need for women to transition into and out of PMTCT services for continued ART in many settings, there is very limited evidence on optimal transition approaches. Ongoing operational research is required to identify sustainable and acceptable transition approaches and service delivery models that support continuity of HIV care during and after pregnancy.

## 1 INTRODUCTION

Sustained engagement in antiretroviral therapy (ART) services during and after pregnancy is critical for the prevention of mother‐to‐child transmission (PMTCT) of HIV and to optimize maternal health. High rates of loss to follow‐up from ART services have been noted among pregnant and postpartum women living with HIV(WLHIV) [1, 2].

**Figure 1:**
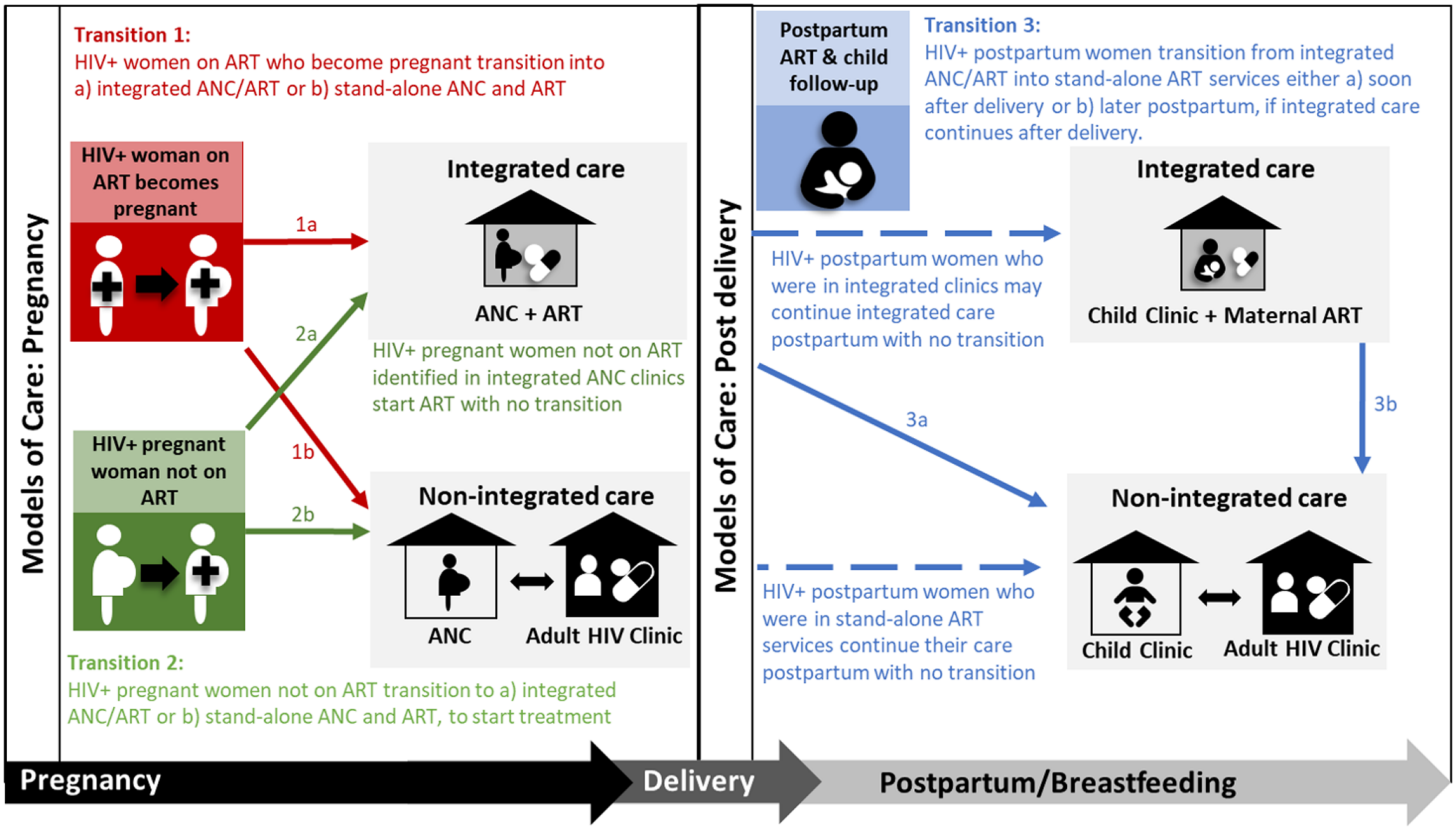
Transition points, models of care and timing of transition into and out of PMTCT for pregnant and postpartum women. Dashed lines represent continued care with no transition needed. ART, antiretroviral therapy; ANC, antenatal care.

The first programmatic guidance for ART for women’s own health was released by the World Health Organisation (WHO) in 2006 [3]. This prompted the scale‐up of the provision of ART to women diagnosed with HIV during pregnancy who met the CD4 cell count requirements. If eligible, women were usually referred from antenatal care (ANC) to general ART services for treatment. In addition to missing eligible women due to poor access and delays with CD4 testing, many identified as ART eligible did not successfully transition to initiate ART prior to delivery [4, 5]. Integrated ANC and ART services (ANC/ART) were subsequently recommended, greatly improving the uptake of ART during pregnancy [6]. Similarly, continued integrated maternal ART and child health services postpartum, including early infant HIV diagnosis, have been associated with improved maternal and child health outcomes [7, 8, 9]. In the current context of universal ART, WLHIV should remain on effective ART for life, with the addition of PMTCT and routine maternal health services during periods of pregnancy and breastfeeding. However, even with universal ART and integrated ANC, maternal ART and child health services, most PMTCT services are not designed to keep mothers in care indefinitely and women will transition from PMTCT to general ART clinics at some point after delivery. Women already established on ART when they conceive need to transition into PMTCT services either in a stand‐alone ANC clinic or an integrated ANC/ART clinic for the duration of pregnancy with transition back to general ART care postpartum. A framework to describe the transitions for continued ART among women moving into and out of PMTCT is depicted in Figure 1. This cycle may occur multiple times for WLHIV, particularly in settings with high fertility rates.

Approaches to these transitions are not consistent and few countries have specific guidelines on transitions for HIV care during and after pregnancy [10, 11]. Although integrated ANC/ART services for pregnant women are recommended by the WHO [12], this model has not been adopted in all countries and some pregnant women still attend separate ART and ANC clinics. Furthermore, the timing of postpartum transition from PMTCT to general HIV services is variable and ranges from about six to ten weeks postpartum in South Africa and parts of Malawi, to up to two years postpartum in Kenya, Zimbabwe and Mozambique [11, 13, 14, 15, 16, 17, 18]. Successful transition and continuity of care for women on ART is critical to ensure their long‐term health, however, it is not clear how this step in the HIV care cascade is addressed in many settings.

This review aimed to synthesize the available evidence on existing approaches to transitioning women into and out of PMTCT services for continued ART. We examined approaches described in the literature and the evidence for which transition approaches improve outcomes of linkage to ART initiation, linkage to continued ART or retention in care following a transition. In addition, we examined factors that may influence successful transition into or out of PMTCT services.

## 2 METHODS

This review was conducted following the Preferred Reporting Items for Systematic Reviews and Meta‐Analyses (PRISMA) guidelines [19]. A comprehensive electronic search was conducted online in PubMed and SCOPUS. Conference abstracts from major international HIV‐focused meetings (Conference on Retroviruses and Opportunistic Infections, International AIDS Society Conference on HIV Science, International AIDS Conference, International Workshops on HIV Pediatrics, Women and HIV, Adolescents and HIV) were also searched, but none were suitable for inclusion.

Search terms and MeSH (medical subject heading) equivalents were searched using the search strategy: (HIV OR “human immunodeficiency virus”) AND (“Antiretroviral therapy” OR ART OR “antiretroviral treatment” OR “anti‐retroviral therapy” OR antiretroviral OR ARV OR “prevention of mother‐to‐child transmission” OR PMTCT OR “HIV care”) AND (pregnancy OR pregnant OR antenatal OR perinatal OR maternal OR postpartum OR postnatal) AND (transition OR transfer OR refer OR link OR linked OR linkage OR “model of care” OR integrated OR integration OR cascade OR continuum).

English language publications from January 2006 through July 2020 were included with no restrictions placed on study design or geographic location. While 2003 WHO guidance included pregnant and breastfeeding women, scale‐up of lifelong ART in the context of PMTCT was not included until 2006 [3]. Studies launched in 2005, but with most data collection in the review period were also included. Studies that did not contain information or data specific to the transition of women into or out of PMTCT services were excluded. As such, studies reporting on outcomes in integrated services with no transition were not included. Investigators were not contacted for further details. Letters, editorials, commentaries and review articles were excluded, but reference lists were checked to identify missed papers. Where more than one published paper described the same study cohort, multiple papers were included if unique information was presented in each, otherwise only the paper with the most complete data on transition approach was included.

Titles and abstracts of the studies were merged and de‐duplicated in Mendeley and screened independently by two reviewers. Full texts of all studies meeting inclusion criteria were reviewed independently by both reviewers. Reviewer disagreement was resolved through third party discussion, as needed.

Studies not focused on transition, but that described an approach to transition in the methods or results were also included. Models of care transitioned between (stand‐alone ART vs. ART integrated ANC, HIV and/or childcare) were also extracted. Since most studies included were not primarily focused on transition but only described transition or outcomes after transition in the context of other primary research questions, the quality of evidence was not included in this review.

Through discussion and consensus with all authors, three possible transition points into or out of PMTCT services for continued maternal ART were defined (Figure 1). The location of childcare is included in the figure; while not the focus of this review, it is strongly linked to maternal postpartum HIV care. Transition 1 is when women on ART in general HIV services transition into integrated ANC/ART or stand‐alone services when they conceive. Transition 2 is when pregnant women not yet on ART (either newly diagnosed or known HIV‐positive) transition into either integrated ANC/ART services or stand‐alone ART services to initiate treatment. Transition 3 is after delivery when women who have been in integrated ANC/ART services transition into general ART services either soon after delivery or later postpartum if integrated care is continued after delivery. Depending on the model of care, a transition may not be needed. For instance women in non‐integrated care during pregnancy remain in stand‐alone ART services postpartum.

The number of studies reporting on transitions at each of these points was enumerated and results were grouped and described as follows:

### 2.1 Description of transition approaches

All studies that described approaches to transition into or out of PMTCT services for ART were included and thematically grouped into similar strategies. Outcomes following a point of transition (where reported) were also described. Possible outcomes included: (1) successful linkage to care after transition, (2) ART initiation during pregnancy after transition from ANC to general ART services (transition 2 only) and (3) retention in care after transition.

### 2.2 Comparison of outcomes between two or more transition approaches

We included all studies that compared any of the above‐mentioned outcomes after the transition between two or more transition approaches, including proportions of women experiencing the outcome and relative measures of association, where available. Due to the very small number of studies comparing outcomes between different approaches to transition and the heterogeneity in outcome type, timing and definition, no pooled estimates were calculated.

### 2.3 Factors influencing successful transition

All studies, both quantitative and qualitative, exploring barriers or facilitators to transitioning into or out of PMTCT services were included with factors grouped by theme. We described factors reported to influence the transition process or successful transition, but factors related to other outcomes such as ART initiation and retention were excluded.

## 3 RESULTS

**Figure 2:**
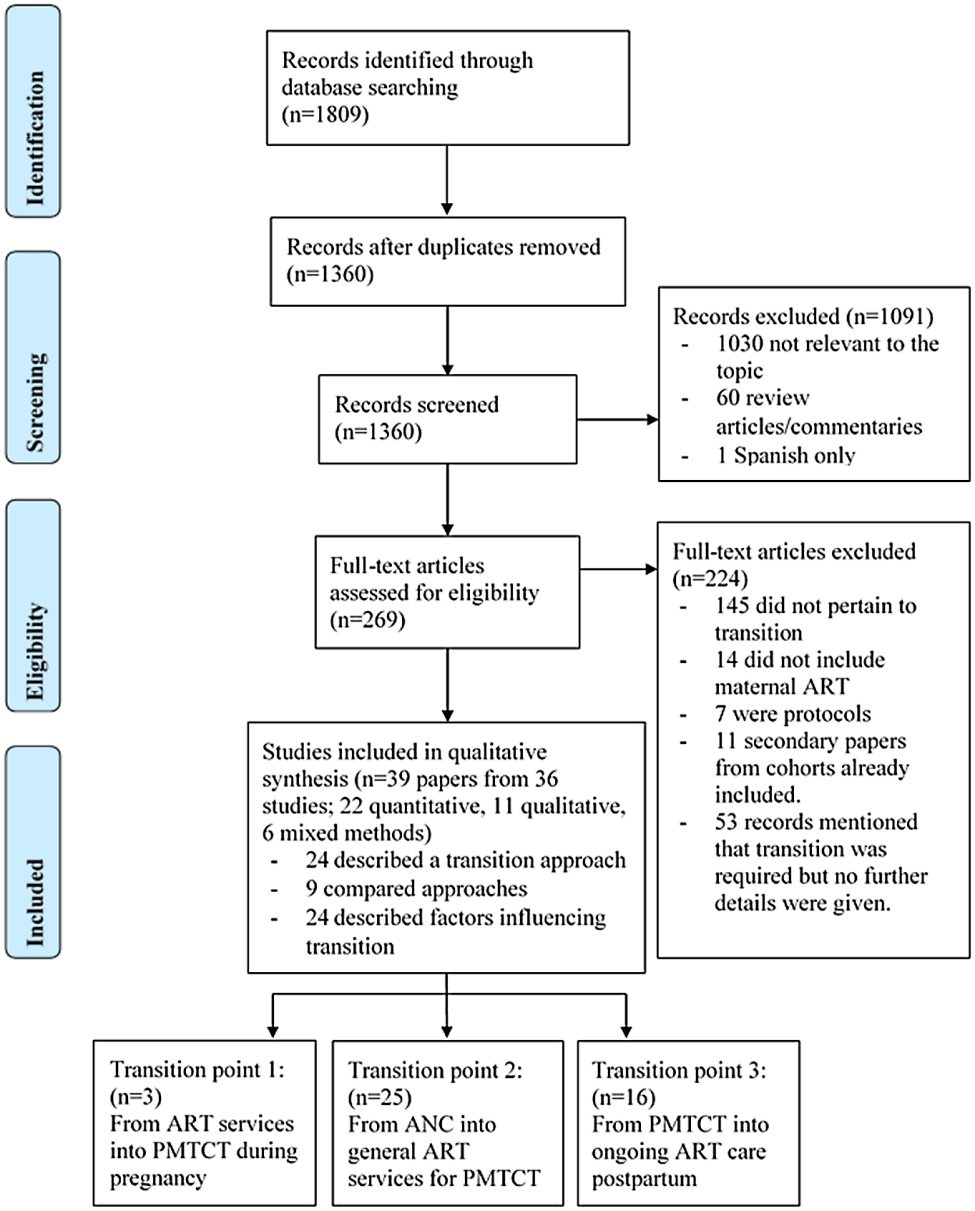
PRISMA flow diagram summarising the literature search. Note each included study may appear in multiple results groups and transition points. ANC, antenatal care; ART, antiretroviral therapy; PMTCT, prevention of mother‐to‐child transmission of HIV.

The initial search located 1809 abstracts (Figure 2). After removing duplicates (n = 449), review and commentary articles (n = 60) and unrelated titles and abstracts (n = 1030), 269 full‐text articles remained. After review, 224 were excluded as they did not pertain to transition into or out of PMTCT for ART, were study protocols, or were secondary papers from cohorts already included. Among these were 53 papers that mentioned that a transition took place, but did not present relevant data. Thus, 39 papers met the inclusion criteria: 22 quantitative, 11 qualitative and 6 mixed methods studies. Two studies reported relevant transition data in two or more papers; thus 36 unique studies were included.

A summary of all included studies is presented in Table S1, arranged by the points of transition reported in each study. The study periods ranged from 2005 to 2018 and included data from Botswana, Cambodia, Cote de’Ivoire, Democratic Republic of Congo, India, Kenya, Malawi, Mozambique, Myanmar, Nigeria, Rwanda, South Africa, Tanzania, Uganda, the United States and Zambia. Overall, three studies include transition 1, 25 transition 2 and 16 transition 3. Twenty‐four studies described factors that may influence transition into or out of maternal ART.

### 3.1 Description of transition approaches

In 24 studies that described a transition approach, the approaches were grouped broadly into (1) information provided at the point of transition (transfer letters and counselling), (2) strengthened communication or data linkage between services, (3) transition navigators or (4) a combination of approaches. The approach description along with outcomes after transition (where reported) is presented in Table 1.

**Table 1:** Description of approaches to transitioning women into or out of PMTCT for continued ART (24 studies)

	First author, year	Study period	Location	Description of transition approach	Linked to the new clinic after any transition	Initiated ART (only for transition point 2)	Retained in HIV care after any transition
Information given at the point of transition (transfer letters and counselling or instructions)								
Transition point 2	Stinson, 2013 [20]	2008	South Africa, urban (three clinics, 658 women)	Pregnant women eligible for ART in ANC were provided with a transfer letter to either the adult HIV clinic on the same premises as ANC or to a distal, stand‐alone HIV clinic	Not reported	‐ 38% of ART eligible women start ART during pregnancy on the same premises ‐ 45% of ART eligible women start ART during pregnancy of those who went at a stand‐alone ART clinic	Not reported
	Mugasha, 2014 (rural group) [21]	2012	Uganda, rural (three clinics, 267 women)	Pregnant women eligible for ART in a rural ANC clinic were provided with a transfer letter and given directions to the adult HIV clinic	25% enrolled in the HIV clinic by six weeks postpartum	Not reported	Not reported
	Price, 2014 [22]	2011 to 2013	Malawi, rural (nine clinics, 395 women)	Pregnant women in clinics not offering integrated ANC and ART were provided with a transfer letter to an adult HIV clinic	Outcomes not disaggregated by women who transferred to an ART clinic and those who received integrated care	Outcomes not disaggregated by women who transferred to an ART clinic and those who received integrated care	Not reported
	Myer, 2015 (standard of care group) [23]	2010 to 2013	South Africa, urban (one clinic, 1615 women)	Pregnant women eligible for ART in ANC were transferred to their nearest adult HIV clinic with a transfer letter and counselling to attend the clinic	Not reported	23% initiated ART during pregnancy	Not reported
Transition point 3	Otieno, 2010 [24]	2005	Kenya, urban (four clinics, 239 women)	Women who had received integrated ANC and ART during pregnancy, and integrated childcare and ART postpartum during breastfeeding were provided with a transfer letter to attend their nearest adult HIV clinic at two years postpartum	74% reported attending the transfer clinic (median 17 months post‐transfer)	NA	63% of those who linked to the new clinic retained at a median of 17 months after the transition
	Phillips, 2015 [13]	2013	South Africa, urban (one clinic, 279 women)	Women who had received integrated ANC and ART during pregnancy were transferred to their nearest adult HIV clinic at six weeks postpartum with a standard transfer letter and counselling on attending the ART clinic	74% linked to an ART clinic within a year of transfer	NA	Not reported
	Cheshi, 2019 [25]	2014	Nigeria (two hospitals, 372 women)	Women were referred from PMTCT to continue ART at ART services by either written referral letter or oral referral. The referral may have been done by a doctor, nurse or counsellor	15% of women did not link to ART services	Not applicable	Not reported
	Phillips, 2018 [26] and Phillips, 2020 [27]	2013 to 2016	South Africa, urban (one clinic, 617 women)	Women who had received integrated ANC and ART during pregnancy were either transferred to an adult HIV clinic at six weeks postpartum or they received integrated childcare and ART until cessation of breastfeeding and then were transferred to an adult HIV clinic. At transfer women received a standard transfer letter.	79% linked to continued ART care (82% of those transferred six weeks postpartum and 74% of those transferred after cessation of breastfeeding)	NA	75% of those who linked to a new clinic retained through 24 months on ART (59% in care through 24 months overall) 57% and 45% experienced no 180‐day gap in care by 36 months postpartum
Strengthened communication or data linkage between services								
Transition point 1	Rawizza, 2015 [28]	2015	Nigeria (31 clinics, 31 504 women)	Pregnant women who were established on ART and became pregnant had their ART identifier, maternal PMTCT identifier and baby identifier linked to facilitate linkage between different services.	Not reported	Not reported	Not reported
Transition point 2	Gupta, 2016 [29]	2014	India, urban and rural (578 testing sites, 70 HIV clinics, 1118 women)	Pregnant women who tested HIV positive in ANC were entered into an electronic line listing to track service attendance through pregnancy and up to 18 months postpartum. Each testing centre (578 included) had their own listing of pregnant women which was shared with the HIV clinics (70 included).	Not reported	91% of women started ART in pregnancy.	Not reported
	Kyaw, 2019 [30]	2012 to 2017	Myanmar, urban (five clinics, 303 women)	A nurse allocated as PMTCT focal person for Township Health Department and a medical doctor assigned by the district as responsible for PMTCT and for documenting linkage to care. The doctor collects a weekly list of women newly registered from the PMTCT focal person and searches for them in the electronic HIV clinic databases to ensure linkage.	Not reported	84% of women started ART. Median 20 (10 to 51) days between diagnosis and referral.	Not reported
Transition navigator								
Transition point 1 and 2	Geldsetzer, 2019 [31]	2012 to 2014	Tanzania, urban (36 wards)	Community health workers actively identify pregnant women in the community through home visits, refer to ANC and PMTCT, and follow‐up to check if women linked to care.	Results were not disaggregated for women living with and without HIV	Not reported	Not reported
Transition point 2	Stover, 2019 [32]	2014 to 2015	Mozambique (39 communities)	Community improvement team set up to share information about antenatal care and actively identify pregnant women in the community. The community group provides a referral form and informs the antenatal clinic when a woman is identified who then monitor whether she has attended or not.	Results were not disaggregated for women living with and without HIV	100% of women initiated ART. No change in ART initiations over time	Not reported
	Tsague, 2010 [33]	2006 to 2008	Rwanda (urban and rural)	Pregnant women eligible for ART in ANC were either escorted to the adult HIV clinic on the same premises as ANC or were transported to a stand‐alone adult HIV clinic.	‐ 67% of those transferred to clinic on the same premises ‐ 35% of those transferred to a stand‐alone clinic	‐ 78% of women eligible for ART and transferred to the clinic on the same premises ‐ 85% of those eligible for ART and transferred to a stand‐alone clinic	Not reported
	Ferguson, 2014 [5]	2010	Kenya, urban (1 district hospital, 100 women)	Pregnant women eligible for ART in ANC were accompanied by the ANC nurse to the adult HIV clinic on the same premises as ANC.	53% of women registered at the HIV clinic	Not reported	Not reported
	Myer, 2015 (enhanced linkage group) [23]	2010 to 2013	South Africa, urban (one clinic, 1615 women)	Pregnant women eligible for ART in ANC were accompanied by a patient navigator to enrol at the adult HIV clinic on the same premises as ANC.	Not reported	45% initiated ART during pregnancy	Not reported
	Kojima, 2017 [34]	2008 to 2011	India, rural (three mobile and 1 standing clinic)	Pregnant women eligible for ART in ANC were accompanied by a health provider to the tertiary care facility where ART was provided.	Not reported	Not reported	Not reported
	Suryavanshi, 2018 [35]	2015 to 2016	India, urban and rural (four districts, 60 outreach workers)	Pregnant women eligible for ART in ANC were accompanied by outreach workers to the adult HIV clinic. The outreach workers also provided appointment reminders and follow‐up through 18 months postpartum.	Not reported	Not reported	Not reported
Transition point 3	Hackett, 2019 [36]	2011 to 2017	USA, urban (one clinic, 275 women)	A multidisciplinary perinatal care coordination team meet monthly to determine case plans for women from prenatal care through transition to HIV care postpartum. Specifically: ‐ Strengthen direct communication and referral between PMTCT and HIC services ‐ To improve appoint scheduling at the HIV service ‐ To provide psychosocial and clinical support	‐ 79% of women linked to HIV care within 90 days of delivery following implementation of the perinatal care coordination team ‐ Prior to implementation of this approach, 29% linked to HIV care within 90 days of delivery		‐ 55% of women were retained at 12 months postpartum following implementation of the perinatal care coordination team ‐ Prior to implementation of this approach, 45% of women were retained at 12 months postpartum
Transition point 2 and 3	Killam, 2010 [37]	2007 to 2008	Zambia, urban (eight clinics, 1566 women)	Women eligible for ART in ANC were either transitioned to an adult HIV clinic in pregnancy or received integrated ANC and ART with transition to an adult HIV clinic at six weeks postpartum. At the transfer, women were escorted by a peer educator to the ART clinic on the same premises as the ANC clinic. Outcomes after transition not assessed in the integrated care group.	Only assessed among women who transitioned during pregnancy, 25% of whom enrolled in the HIV clinic	‐ 14% of women transferred during pregnancy initiated ART ‐ 33% of women who received integrated ANC and ART.	Among women who started ART, 91% of women who had transitioned during pregnancy were retained after 90 days compared to 88% of women who received integrated ANC and ART.
	Anderson, 2017 [38]	2005 to 2013	USA (849 births)	Voluntary enrolment in a perinatal case management program. Medical case managers assist with navigating the complex health system and provide psychosocial support.	42.9% linked to HIV care within 90 days of delivery	Not reported	52.7% retained in care at one year postpartum
All transition points	Besada, 2018 [39]	2015	Cote D’Ivoire, DRC, Malawi, Uganda (urban and rural)	Community cadres were employed to assist pregnant and postpartum women with navigating to the required pregnancy, PMTCT and postnatal services across any required transitions.	Not reported	Not reported	Not reported
Combination of approaches								
Transition point 2	Weigel, 2012 [40]	2006 to 2009	Malawi, urban (one hospital and two clinics, 612 women)	Pregnant women eligible for ART during ANC were transferred to adult HIV clinics. Over time the following approaches were employed: ‐ Referral letter and monitoring to ensure linkage ‐ Providing transport to the ART clinic ‐ Patient held “health passports” ‐ Memorandum of understanding between ANC and ART services to clarify roles and strengthen continuity of care ‐ Priority given to pregnant women arriving at the ART clinic to be seen quickly ‐ Information leaflet with directions and details for the ART clinic ‐ Signposts from the antenatal clinic to the ART clinic ‐ Encouraging flexibility about which ART clinic is attended ‐ Linked electronic data systems	‐ 56% of women reached the ART clinic. ‐ Reduced time to link to the ART clinic from 41 days in 2006 to 15 days in 2009. ‐ Did not report on outcomes for each intervention.	‐ 47% of women started ART during pregnancy. ‐ Improved ART initiation during pregnancy from 17% in 2006 to 74% in 2009.	‐ Increased retention at six months after ART initiation (of all eligible women) from 17% in 2006 to 65% in 2009.
	Mugasha, 2014 (urban group) [21]	2012	Uganda, urban (two clinics, 743 women)	Pregnant women eligible for ART in urban ANC clinics were transferred to an adult HIV clinic. They were: ‐ Provided with a transfer letter ‐ Received guidance from a peer mother on transitioning to the ART clinic	41% enrolled in the HIV clinic by six weeks postpartum	Not reported	Not reported
	Saleem, 2014 [41]	2012	Uganda (11 clinics, 48 women, 11 providers)	Pregnant women eligible for ART in ANC were transferred to adult HIV clinics with: ‐ Written referral letter ‐ Verbal counselling on transfer ‐ Provider escorted women from ANC to the HIV clinic	Not reported	Not reported	Not reported
Transition point 3	Myer, 2017 [42]	2015	South Africa, urban (one clinic, 129 women)	Women who had received integrated ANC and ART during pregnancy were transferred to an adult HIV clinic soon after delivery. They were: ‐ Counselled on the transfer process and given a standard transfer letter. ‐ Accompanied by a PMTCT counsellor to enrol in the adult HIV clinic on the same premises as ANC	Not reported	Not applicable	Not reported

If studies had multiple arms, we have only included here the arms or components that required a transition. ANC, antenatal care; ART, antiretroviral therapy; NA, not applicable; PMTCT, prevention of mother‐to‐child transmission of HIV.

### 3.2 Information provided at the point of transition

Eight studies described the use of letters to facilitate transition: four from ANC to HIV services to start ART (transition 2) in South Africa [20, 23], Malawi [22] and Uganda [21], and four from integrated ANC/ART services to a general ART clinic after delivery (transition 3) in South Africa [13, 26], Nigeria [25] and Kenya [24]. None provided the content of the transfer letter. Three studies reported that counselling or instructions on the transition process were provided at the point of transition with few details on pre‐transition counselling. In Nigeria, women received a written or a verbal referral from either a doctor, nurse, counsellor or laboratory staff [25]. Overall, 15% of women did not link to continued ART postpartum. The Ugandan study specified that, in addition to a transfer letter, women transitioning to an HIV clinic to start ART in a rural area were given directions to the clinic [21]. In this study, 25% of women successfully transitioned to the general ART clinic to initiate ART before six weeks postpartum. One Kenyan [24] and two South African [13, 26] studies assessing transition 3 reported 74% to 79% of women successfully linked from PMTCT to general ART clinics to continue care postpartum. Among women who successfully linked to the ART clinic, 63% was retained at a median of 17 months after transition in the Kenyan study [24] and 75% was retained through 24 months on ART in South Africa [26]. In a follow‐up to the South Africa study reporting on retention overall, 45% to 57% of women remained in care with no gap through 36 months postpartum [27]. ART initiation during pregnancy after transition 2 ranged from 23% to 45% following transition from ANC to a general ART clinic with a transfer letter and counselling provided at the point of transition in two South African studies [20, 23].

### 3.3 Strengthened communication or data linkage between services

Linking patient data between different services was used to assist the transition between clinics in studies in India [29], Myanmar [30] and Nigeria [28]. In Myanmar, direct linkage was established through a PMTCT focal nurse for ANC facilities and a district PMTCT doctor assigned to document linkage to HIV care during pregnancy [30]. During the study period 84% of women started ART with the median 20 days between diagnosis and referral. In India, a stand‐alone electronic line‐listing of all WLHIV presenting for ANC was shared between clinics to facilitate tracing of women through pregnancy and up to 18 months postpartum [29]. In Nigeria, pregnant women already enrolled on ART received unique PMTCT patient identifiers to allow records to be linked across all services [28]. PMTCT identifiers were linked to adult ART and infant patient identifiers in the electronic medical record.

### 3.4 Transition navigators

There were 11 studies describing the use of transition navigators conducted in India [34, 35], South Africa [23], Rwanda [33], Zambia [37], Tanzania [31], Mozambique [32], Kenya [5], the United States [36, 38], and a multisite study in Cote D’Ivoire, Democratic Republic of Congo, Malawi and Uganda [39]. Studies from Kenya and India described an approach where healthcare providers escorted women from ANC to HIV clinics which were either on the same premises or nearby [5, 34]. Other studies described the navigator as a “peer educator” [37], “patient navigator” [23], “outreach worker” [35] or “lay health worker” [39] who held additional responsibilities including counselling, sending appointment reminders, following‐up with women, and community engagement. In Rwanda, women diagnosed with HIV in ANC were escorted to either an ART clinic on the same premises or a general ART clinic with transport provided [33]. Linkage to care after transition from ANC to HIV services ranged from 25% in Zambia [37], 53% in Kenya [5] and 67% in Rwanda [33]. Most did not report retention outcomes however in Zambia retention was high among women who successfully started ART (91% at 90 days after ART initiation).

Two studies described community‐based approaches to actively identify pregnant women in the community and ensure linkage to antenatal and HIV care [31, 32]. The Tanzanian study did not report outcomes for WLHIV specifically, but in Mozambique, 100% of women identified by the community team successfully initiated ART [32]. Two US studies described the implementation of case managers [38] and a perinatal care coordination team [36] to strengthen transition from PMTCT during pregnancy to postpartum HIV care. Using voluntary enrolment into a case management programme, 43% of women linked to HIV within 90 days of delivery and 53% was in care one year postpartum [38]. Following the implementation of a multidisciplinary care coordination team, 79% of women was linked to care within 90 days of delivery [36].

### 3.5 Combination approaches

Four studies described the use of a combination of transition approaches. Two Ugandan studies examined the combination of a transition navigator with counselling and a transfer letter to facilitate transition 2 [21, 41]. A South African study of postpartum women transitioning to ART services after delivery described women being accompanied to the nearby ART clinic by a PMTCT counsellor, plus counselling and a transfer letter [42]. Lastly, a study in Malawi described the evolution of approaches to transitioning pregnant women from ANC to ART care to start treatment [40], including transfer letters, transport provision to new clinic, patient held care record, information leaflets with directions and signposting for the ART clinic, a memorandum of understanding between the ANC and the ART clinics to clarify roles, and linked health information systems. The incremental value of each approach was not assessed, but from 2006 to 2009, the investigators observed a reduction in median time to linkage to the ART clinic (from 41 to 15 days), increased proportions of pregnant women successfully initiating ART, from 17% to 74%, and improved six‐month retention after ART initiation, from 17% to 65% [40].

Although outcomes of different transition approaches varied substantially and there was heterogeneity in study methods and reported outcomes, successful linkage to an ART clinic to start treatment in pregnancy (transition 2) ranged from 25% using transfer letters [21] or peer navigation [37], to 56% using a combination of approaches [40] and 67% with escort to the ART clinic on the same premises as ANC [33]. Successful linkage to general ART services from PMTCT postpartum (transition 3) was only reported in studies employing a transfer letter and counselling approach with over 70% of women linking to ongoing care in all three studies [13, 24, 26].

### 3.6 Comparison of outcomes between two or more transition approaches

Seven studies compared outcomes between two or more transition approaches (Table 2), four focused on approaches to transition point 2 (ANC into ART services during pregnancy) [20, 21, 23, 33] and three focused on transition 3 [25, 36, 38]. In Rwanda, women were escorted from ANC to either an ART clinic on the same premises or a stand‐alone ART clinic (transport provided) [33]. Women transferred to the clinic on the same premises were almost twice as likely to successfully link (67% vs. 35%; risk ratio [RR] 1.9 95% CI 1.5 to 2.3). A South African study in which only a transfer letter was provided compared ART initiation among pregnant women eligible for treatment transferred to an ART clinic on the same premises (38%), to those transferred to a more distal ART clinic (45%), and to those receiving ART integrated in ANC (55%, global p = 0.003) [20].

**Table 2:** Summary of studies comparing two or more approaches to transition women into or out of PMTCT for ART services

First author, year	Study period	Location	Transition models/approaches	Outcome definition	Results
Transition point 2 (HIV + pregnant women not on ART transition to integrated ANC/ART or stand‐alone ART and ART services to start treatment)					
Tsague, 2010 [33]	2006 to 2008	Rwanda (urban and rural)	Transferred to the ART clinic on the same premises at the antenatal clinic, women were escorted to the clinic. Transferred to a stand‐alone ART clinic with an escort and transport was provided	Enrolment in the ART clinic	Pregnant women transferred to the HIV clinic on the same premises were twice as likely to enrol at the ART clinic compared to those transferred to a stand‐alone ART clinic (RR 1.9, 95% CI 1.5 to 2.3)
Stinson, 2013 [20]	2008	South Africa (urban)	Integrated antenatal ART one day a week Proximal – referred within 100m Distal – referred within 5km All women were given a referral letter with no follow‐up to ensure linkage	ART initiation during pregnancy	The proportion of ART eligible women who initiated ART in pregnancy was 55% in the integrated antenatal/ART clinic, 38% when referred to a proximal ART clinic, and 45% when referred to a distal ART clinic (p = 0.003)
Mugasha, 2014 [21]	2012	Uganda (urban and rural)	Referral letter and guidance from a peer mother (in urban facilities) Referral letter and provided with direction to the ART clinic (rural facilities)	Linkage to HIV care before six weeks postpartum	The proportion of pregnant women who linked to the HIV clinic was 41% if the urban group and 25% in the rural group.
Myer, 2015 [23]	2010 to 2013	South Africa (urban)	Provided with a referral letter Accompanied by a patient navigator Integrated services, no antenatal transition required	ART initiation during pregnancy	The proportion of women who initiated ART in pregnancy was 23% in the group that only received a transfer letter and 45% in the group that was accompanied by a patient navigator. 78% of women successfully initiated ART in the integrated antenatal ART group (p < 0.001).
Transition point 3 (HIV + postpartum women transition from integrated PMTCT into non‐integrated ART services soon after delivery or later postpartum)					
Cheshi, 2019 [25]	2014	Nigeria (urban and rural)	Oral versus written referral Referring provider: Doctor versus nurse, lab staff or counsellor	Linked to ART service within six months after referral from PMTCT.	No difference in successful linkage between oral and written referral (OR 1.3 95% CI 0.8 to 2.4). Referral by a doctor increased the odds of successful linkage compared to referral by a nurse, counsellor or lab staff (OR 2.0 95% CI 1.0 to 4.0)
Hackett, 2019 [36]	2011 to 2017	USA (urban)	Standard referral between PMTCT and outpatient HIV care postpartum Perinatal care coordination team strengthened communication and referral between PMTCT and HIV providers, streamlined HIV clinic enrolment and scheduling, and provided psychosocial and other support.	Link to outpatient HIV care within 90 days of delivery. Retention in care 12 months postpartum	79% of women who were part of the perinatal care coordination team approach linked to care within 90 days of delivery compared to 29% of women prior to implementation (aOR 9.46 95% CI 4.46 to 20.02) Retention at 12 months was not significantly higher after implementation (55%) compared to before implementation (45%). aOR 1.35 95% CI 0.68 to 2.71.
Anderson, 2017 [38]	2005 to 2013	USA	Had a perinatal case manager Did not have a perinatal case manager	Linked to HIV care within 90 days of delivery Retention at one year postpartum	42.9% of women with a case manager and 35.4% of women with no case manager linked to HIV care within 90 days of delivery (OR 1.36 95% CI 1.03 to 1.80; aOR 1.21 95% CI 0.88 to 1.65) 52.7% of women with a case manager and 34.2% of women with no case manager were retained 12 months postpartum (OR 2.06 95% CI 1.56 to 2.71; aOR 1.59 95% CI 1.17 to 2.16)
Phillips, 2020 [27]	2013 to 2018	South Africa (urban)	Transferred to an adult HIV clinic at six weeks postpartum Received integrated childcare and ART until cessation of breastfeeding and then transferred to an adult HIV clinic.	Experiencing a gap in care (180 days with no evidence of HIV care) after transition and after delivery.	Similar trajectories of time to 180‐day gap after transition in both groups (log‐rank p = 0.068) with gaps frequently occurring soon after transition. By 36 months postpartum, 57% (95% CI 51 to 64%) of women transferred out at six weeks had had a gap in care compared to 45% (95% CI 39% to 52%) of women transferred after weaning.
Transition points 2 (HIV + pregnant women not on ART transition to integrated ANC/ART or stand‐alone ART and ART services to start treatment) and 3 (HIV + postpartum women transition from integrated PMTCT into non‐integrated ART services soon after delivery or later postpartum)					
van Lettow, 2014 [15]	2012 to 2013	Malawi (urban and rural)	Antenatal referral to start ART Start ART in the antenatal clinic but transition at six weeks postpartum to continue ART	Retention six and twelve months after ART initiation, among women who had initiated ART	Retention six months after ART initiation: 80% of women who started ART after antenatal referral to an ART clinic were retained, compared to 89% of women who started ART within ANC and transferred later. Retention 12 months after ART initiation: 80% of women who started ART after antenatal referral to an ART clinic were retained, compared to 87% of women who started ART within ANC and transferred later.

ANC, antenatal care; ART, antiretroviral therapy; NA, not applicable; PMTCT, prevention of mother‐to‐child transmission of HIV.

Another South African study compared ART initiation among pregnant women transferred to a nearby ART clinic using either transfer letters or patient navigators to ART initiation among pregnant women in integrated ANC/ART with no transition [23]. Among women with patient navigators, 45% of ART‐eligible women initiated treatment during pregnancy compared to 23% with transfer letters (hazard ratio [HR] 3.05 95% CI 2.11 to 4.40), but the highest ART initiation was among women in integrated care, among whom78% initiated ART and who were seven times more likely to start ART compared to women with just transfer letters (HR 7.6 95% CI 5.5 to 10.4). A study in Uganda in 2012 compared linkage to HIV care from ANC after transition in rural and urban areas [21]. In urban areas where women received a transfer letter and were accompanied by a peer navigator, 41% successfully linked to the HIV clinic, compared to 25% in rural areas where women received a referral letter and instructions on how to get to the ART clinic without a peer navigator.

At transition 3, a Nigerian study found no difference in linkage to ART within six months of postpartum referral among women who received a written versus verbal referral [25]. Women referred by a doctor had two‐fold higher odds of successful linkage compared to those referred by a nurse, counsellor or laboratory staff (OR 2.0 95% CI 4.46 to 20.02). Women who had a perinatal case manager in the United States were slightly more likely to link to HIV care within 90 days postpartum (42.9% versus 35.4%, OR 1.36 CI 1.03 to 1.80) and significantly more likely to be retained at 12 months postpartum (52.7% vs. 34.2%, OR 2.06 95% CI 1.56 to 2.71) compared to women with no case manager [38]. Also in the United States, the implementation of a perinatal care coordination team increased linkage to HIV care within 90 days postpartum from 29% to 79% (aOR 9.46 95% CI 4.46 to 20.02) [36].

In addition, one study compared outcomes between transition point 2 and integrated ANC/ART care with postpartum transition to general ART services (transition 3) [15], and another compared early versus late transition from integrated PMTCT to ART services postpartum [27]. A survey of models of care in Malawi compared outcomes of pregnant women after transition 2, to outcomes among women after transition 3 at six weeks postpartum [15]. Although ART initiation was substantially higher when ART was integrated into ANC, retention in ART care at six and twelve months after ART initiation was similar: 89% and 87% of women who transferred postpartum were retained at six and twelve months, respectively, compared to 80% retention at both six and twelve months among women who transferred to start ART during pregnancy [15]. When comparing transition to ART services at six weeks postpartum to transition after cessation of breastfeeding in South Africa, gaps in care were observed soon after transition in both groups with similar trajectories [27]. By 36 months postpartum, 57% of women transferred at six weeks and 45% of women transferred after weaning had experienced a gap in care.

### 3.7 Factors influencing transition into or out of PMTCT services

**Figure 3:**
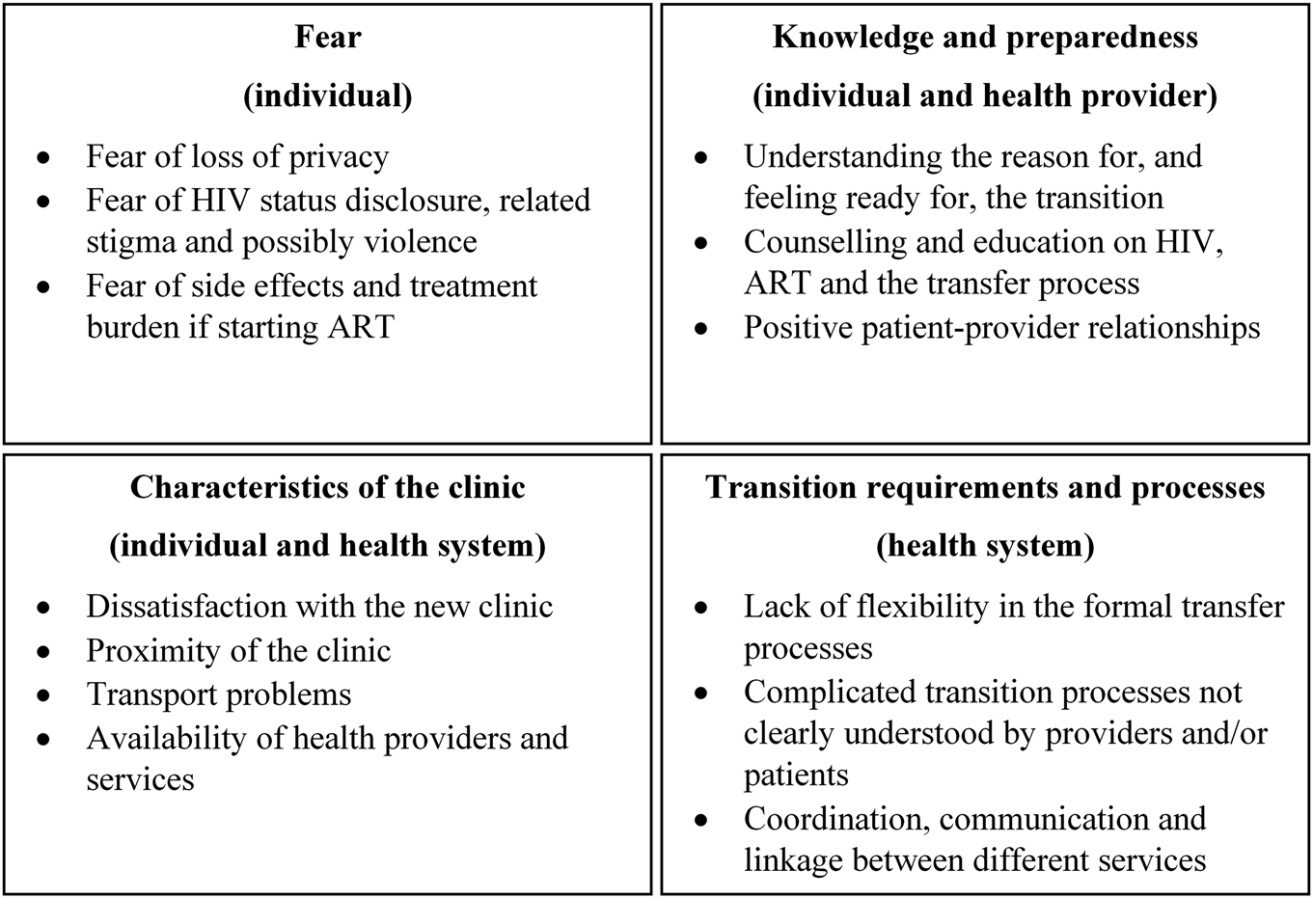
Summary of factors influencing transition into or out of PMTCT for ART services. ANC, antenatal care; ART, antiretroviral therapy; NA, not applicable; PMTCT, prevention of mother‐to‐child transmission of HIV.

Twenty‐four studies reported barriers or facilitators to transition into or out of PMTCT services: 13 focused on transition 2, 9 on transition 3, one on both transitions 2 and 3, and one on transitions 1 and 2. Factors reported were grouped into four themes: fear, knowledge and preparedness, clinic characteristics, and transition process/requirements of the health system (Figure 3).

### 3.8 Fear

Fear was frequently cited by women as a barrier to successful transition between services at transition 2 and 3, including fear of consequences from HIV status disclosure, lack of confidentiality and experiencing stigma when moving to a new clinic [4, 21, 25, 41, 43, 44, 45]. Fear of stigma and disclosure were also associated with not successfully transitioning in quantitative studies [22, 24, 46]. Women in South Africa expressed reluctance to leave integrated PMTCT services to continue ART and fear about needing to transfer [47]. Stigma and fear of disclosure were also highlighted as barriers by providers in the context of linking pregnant adolescents to HIV services in Kenya [48] and as reasons for not using referral letters provided during community identification of pregnant women in Tanzania [31].

Women in two studies reported fear of physical violence from partners if they found out about their HIV status [24, 41], while having adequate support from a partner or family member was reported as an enabler of transition [5, 21]. Women transitioning from ANC to ART clinics to start treatment reported fear of medication side effects and the burden of being on treatment as additional barriers [22, 24, 41].

### 3.9 Knowledge & preparedness

Another common theme reported at both antenatal and postpartum transitions was lack of knowledge and preparedness for the transition. Some women reported not feeling ready and lacking understanding of why they needed to transition to a new clinic [4, 5, 24, 49]. A Nigerian study found 12% of women referred from PMTCT to ART services postpartum did not know why they were being referred [25]. In some studies, poor knowledge of HIV, distrust of ART and cultural beliefs including a preference for herbal treatments were reported as transition barriers [4, 22, 50]. A quantitative study in the United States found that women newly diagnosed with HIV in pregnancy were less likely to link to HIV care in pregnancy than women who already knew their status [51].

The provision of counselling and education around the transition process and other topics relating to HIV care and treatment were reported as facilitators of successful transition by women [24, 41] and healthcare providers [21]. Providers in Uganda noted that the large volume of women requiring care limited their ability to provide adequate counselling before transitioning women from ANC to ART services [21]. Poor communication and bad interactions between patients and providers were also noted as barriers to linkage to ART services in Kenya [5].

### 3.10 Characteristics of the clinic

Some factors reported to influence transition were related to the characteristics of the receiving clinic. Some women expressed dissatisfaction with the chosen clinic, including location and anticipated waiting times (reported at transition 2 [4, 35] and 3 [24]). Even the transition to a separate clinic on the same premises was reported as a barrier to linkage to HIV care and ART initiation during pregnancy [20, 40, 41]. Transport problems including cost and distance were commonly reported as barriers to transition [4, 5, 21, 24, 35, 52, 53]; providing transport or covering costs was found to facilitate successful transition in Kenya [5]. Not having HIV treatment providers available at the clinic every day was another barrier for women starting ART in pregnancy [41]. Access to ANC and HIV care, or child health services and HIV care, in the same clinic and preferably on the same day was reported to be a facilitator of successful transition [41, 47, 54].

### 3.11 Transition requirements and process

The administrative requirements and transition process within the health system were both barriers and facilitators of transition. Some studies lacked details, but reported gaps or inadequacies in the referral process as barriers to transition [22, 24]. Watson‐Jones et al reported that healthcare providers had insufficient knowledge of the guidelines for transitioning women to HIV care during pregnancy (transition 2) in Tanzania and that referral letters were not always provided [4]. A Kenyan study also found that pregnant women transitioning to ART clinics were not enrolled if they did not present with a formal transfer letter which was not consistently provided to them [54]. In the same study, women reported difficulty navigating the required transition process and inadequate directions to new clinics and clinic hours information.

Travel and migration were reported as barriers to linking to HIV care in pregnancy (transition 2) [4, 5] and continuing HIV care postpartum (transition 3) [24]. Women in Kenya reported that they were refused care at HIV clinics if they were not permanent residents in the area [5]. Improved coordination between ANC and ART services was reported to be needed in order to trace patients between services at all points of transition [21, 25, 41, 53]. The ‘Linked Response’ intervention in Cambodia was reported to improve systems to trace women through required transitions of care [53] along with improved communication between community interventions and health facilities [53]. Patients and providers in two Ugandan studies reported that being escorted from ANC to the HIV clinic (transition 2) and the presence of peer mothers was helpful for transition [21, 41].

## 4 DISCUSSION

In the current context of universal ART, all WLHIV should initiate ART and remain on treatment for life. However, this has not eliminated the need to transition the location of care to receive ART services during and after pregnancy. We believe this to be the first systematic examination of the available evidence on the transition of pregnant and postpartum women into and out of PMTCT services for continued ART. It highlights that, while many WLHIV are required to transition into and/or out of ART services during and after pregnancy, there has been very little research into what approaches and strategies can make this critical process more successful. Although some studies have described approaches to transition, there are very limited data rigorously evaluating the impact of strategies, including examinations of different models of care. Much of the existing literature focuses on the transition from HIV diagnosis in ANC to an HIV clinic to initiate ART (transition 2) with less attention paid to postpartum transitions from PMTCT into HIV care (transition 3) and we were only able to find one study focusing on the transition of pregnant women already on ART into PMTCT (transition 1). Limited available research provides insights into the barriers to transition, including individual, healthcare provider and health system factors, which can be used to inform the development of transition approaches and guidelines.

The limited data and heterogeneity between studies precluded pooled analyses, however, it is notable that, among the studies reporting outcomes, many women did not successfully link to a new clinic following the transition. Although the definition of successful linkage varied across studies, it was higher in almost all studies of postpartum linkage to ongoing ART (transition 3) in South Africa (74% and 77%), Kenya (74%) and the United States (29%, 79% and 43%) [13, 24, 26, 36, 38] compared to studies evaluating linkage to initiate ART during pregnancy (transition 2). Studies reporting the transition from ANC to ART services during pregnancy showed the successful linkage from 25% in Zambia and Uganda to 67% in Rwanda [21, 33, 37]. Studies that reported retention in care after a successful transition observed that once women had transitioned to a new clinic, the majority (>60% across studies) were retained, underscoring that the transition is the vulnerable point [15, 24, 26, 40]. Similarly, a study comparing transition point 3 with transition at six weeks postpartum versus after weaning found similar trajectories in time to loss from care in both groups after transition [27].

In studies comparing two or more transition approaches, women transferred to HIV services on the same premises as ANC or within very close proximity were more likely to successfully transition than those transferred to stand‐alone clinics, but were still at risk for loss [20, 33]. The use of health navigators appears to improve linkage and engagement in HIV care among pregnant and postpartum women and other populations [55, 56, 57, 58]. A perinatal care coordination team also increased linkage to continued HIV care postpartum in the United States [36], although the resources required for this approach may be a barrier to implementation in high‐burden settings.

We chose not to restrict this review to the era of Universal ART in order to capture transition approaches that may have been successful for lifelong ART prior to this. It is clear that Universal ART has not removed the need for these transitions, but rather that the points of transition have shifted. Fewer women require transition to initiate ART during pregnancy, but a transition to continued ART care postpartum is likely. The postpartum transition has seldom been included in the literature, likely due to difficulties linking women across clinics and relatively short study follow‐up periods. With the expansion of Option B+ for PMTCT and universal ART for all people living with HIV, there are also increasing numbers of women already on ART at conception. Only one study was identified focusing on the transition from general ART services into ANC and PMTCT (transition 1) with few details of the process [28], whereas three studies described strategies using community health workers to actively identify pregnant women and link them to ANC and HIV care [31, 32] or to assist with the navigation of any transitions of care [39]. None of these studies reported on outcomes following transition. Further research is needed to understand the current practices for providing HIV care to pregnant women already on ART, whether transition into integrated ANC/ART services should be recommended, and how best to approach this transition to ensure continuity of care. Alternative models of care such as the provision of ANC within existing ART services also warrant exploration.

The identified factors influencing transition ranged from psychosocial factors to structural factors relating to the transition process. Fear of social consequences was a commonly cited barrier that has been previously recognized [59, 60, 61]. Women in many parts of the world are dependent on their partners and/or families for both social and economic support. Pregnant women may be particularly hesitant to risk the loss of support and may fear other consequences, such as partner violence, as a result of disclosure of their HIV status [61]. The ANC setting may provide a safe space to access HIV care under the umbrella of pregnancy and child health services and transition to stand‐alone HIV services may be particularly daunting [62, 63]. Integrated ANC, ART and child health services may provide supportive care that can achieve optimal outcomes for women however more research is needed to identify patient‐friendly models of care for pregnant and postpartum WLHIV.

Other barriers and facilitators of transition included characteristics of the health facility and system. Although the WHO recommends interventions using case managers or navigators to enhance linkage between services, a review of PMTCT policy and policy implementation in Malawi, South Africa and Tanzania, found that most Malawian, but few other facilities implemented this and none had explicit guidance in their national policy [11]. There is a clear need for guidelines on the transition process and greater understanding by health providers and clients. Better coordination and communication between health facilities could facilitate successful transition between clinics or services. Linking of data systems between services has the potential to make the transition process more seamless and the tracing of women more efficient [28, 29, 53]. Unfortunately, transition outcomes using health information systems strengthening approaches have not been evaluated to date. In addition, better information should be provided to WLHIV on all aspects of the transition process.

We note several limitations. While very broad search terms were used in two databases, it is possible that relevant papers in other databases were missed. We also did not include protocols or trial registries so ongoing work has not been considered. This review considered maternal transitions for ART and did not go into detail on other integrated services such as family planning, or the exact model of ART care before or after PMTCT. These transitions occur within the context of numerous overlapping challenges women experience [64, 65]. Addressing barriers to transition through optimal transition approaches will not necessarily result in sustained long‐term engagement in HIV care, but it could reduce the overall burden of barriers to engagement in care at a time when women may be particularly vulnerable. As approaches to transition are developed, consideration should be made of other barriers that might be present at the time, for instance transitioning a woman to general ART care immediately after delivery may be hampered by the disruptions of the early postpartum period.

## 5 CONCLUSIONS

In summary, transitions of care are inevitable along the PMTCT care cascade and supporting these transitions is essential for continuity of maternal HIV care. In the context of Universal lifelong ART where women may move into and out of PMTCT multiple times, guidelines to ensure women start and remain on ART through these transitions are needed. This review is the first to examine the evidence base for approaches to transition into and out of PMTCT that are being employed, and it highlights the lack of evidence on optimal approaches to transition. Given the growing numbers of women on ART, the lack of evidence on transitioning pregnant women from general ART into PMTCT is particularly notable. The existing approaches as well as the barriers and facilitators of transition that have been identified can be used to develop more rigorous transition guidelines. In addition, ongoing operational research is needed to investigate service delivery models that fit with women’s lives and facilitate lifelong retention in HIV care.

## COMPETING INTEREST

The authors declare that they have no conflicts of interest.

## AUTHORS’ CONTRIBUTIONS

EJA, CAT, AG and SM conceptualized and designed the review. TKP conducted the initial searches and TKP and PM screened all abstracts and conducted the data abstraction. TKP drafted the manuscript. EJA, CAT, AG, BN, SM and PM critically reviewed and revised the manuscript. All authors read and approved the final manuscript.

## Supporting information


**Table S1.** Summary of 36 studies included in this review, arranged by points of transition discussed: transition 1 only( women on ART in adult services transition into PMTCT services when they become pregnant, n = 1), transition 2 only (women diagnosed with HIV during pregnancy, or known HIV positive but not in ART, transition into ART services, n = 19), transition 3 only (postpartum women transition from PMTCT services into ongoing ART services, n = 10), transition 1 and 2 (n = 1), transition 2 and 3 (n = 4), and all three transitions (n = 1)
