# Supplementary material for: Approaches to transitioning women into and out of prevention of mother‐to‐child transmission of HIV services for continued ART: a systematic review
Source: J Int AIDS Soc. 2020 Dec 29;24(1):e25633. doi: 10.1002/jia2.25633 (PMC7771153; doi:10.1002/jia2.25633)
Supplement: Supplementary file 1 — Table S1. Summary of 36 studies included in this review, arranged by points of transition discussed: transition 1 only( women on ART in adult services transition into PMTCT services when they become pregnant, n = 1), transition 2 only (women diagnosed with HIV during pregnancy, or known HIV positive but not in ART, transition into ART services, n = 19), transition 3 only (postpartum women transition from PMTCT services into ongoing ART services, n = 10), transition 1 and 2 (n = 1), transition 2 and 3 (n = 4), and all three transitions (n = 1) [file JIA2-24-e25633-s001.docx]

**Supplementary Table 1. Summary of 36 studies included in this review, arranged by points of transition discussed: transition 1 only( women on ART in adult services transition into PMTCT services when they become pregnant, n=1), transition 2 only (women diagnosed with HIV during pregnancy, or known HIV positive but not in ART, transition into ART services, n=19), transition 3 only (postpartum women transition from PMTCT services into ongoing ART services, n=10), transition 1 and 2 (n=1), transition 2 and 3 (n=4), and all three transitions (n=1).**

| **Study characteristics** | | | | | | | **Results available in the study** | | |
| --- | --- | --- | --- | --- | --- | --- | --- | --- | --- |
| **First author, year [in text citation]** | **Study period** | **Location and size** | **Setting** | **Study population** | **Study design** | **Aim of the study** | **Transition approach described** | **Comparison of transition approaches** | **Factors influencing transition** |
| **Transition point 1 (****HIV+ women on ART who become pregnant transition into integrated ANC/ART or non-integrated ANC and ART services): 1 study** | | | | | | | | | |
| Rawizza, 2015[28] | 2015 | Nigeria (31 clinics, 31 504 women) | Not specified | Pregnant women living with HIV enrolled in PMTCT (median age 30 IQR 27-34) | Retrospective cohort | Evaluated loss to follow-up in PMTCT | yes |  |  |
| **Transition point 2 (HIV+ pregnant women not on ART transition to integrated ANC/ART or stand-alone ART and ART services to start treatment): 19 studies** | | | | | | | | | |
| Dryden-Peterson, 2015[52] | 2009-2012 | Botswana (6 clinics, 10 681 women) | Rural, urban | Pregnant women living with HIV (median age 29 IQR 25-33) | Retrospective cohort/ surveillance | Evaluated the impact of transitioning from Option A to Option B on the risk of mother to child transmission |  |  | yes |
| Ferguson, 2014[5] | 2010 | Kenya (1 district hospital, 100 women) | Peri-urban | Women diagnosed with HIV in pregnancy (aged 16 years and older) | Prospective cohort and in-depth interviews | Quantified uptake of long-term HIV services among HIV positive pregnant women and explore reasons for attrition | yes |  | yes |
| FitzHarrris, 2017[51] | 2013 | USA (61 CDC funded health departments, 138 women) | Rural, urban | Pregnant women living with HIV (aged 15-44 years) | Retrospective cohort | Review linkage to HIV treatment of pregnant women living with HIV |  |  | yes |
| Gupta, 2016[29] | 2014 | India (578 testing sites, 70 HIV clinics, 1118 women) | Rural, urban | Pregnant women living with HIV (age range <20 to >40 years) | Retrospective cohort | Described an excel-based individual tracking tool to aid tracing and follow-up of HIV-infected pregnant women | yes |  |  |
| Helova, 2017[54] | 2014 | Kenya (4 clinics, 40 women, 30 providers) | Rural | Pregnant or postpartum women living with HIV, male partners, and health providers (age 18 years and older) | In-depth interviews and focus groups | Explored challenges to the provision of Option B+ at the health facility level |  |  | yes |
| Kojima, 2017[34] | 2008-2011 | India (3 mobile and 1 standing clinic) | Rural | Pregnant women living with HIV (age not specified) | Prospective cohort | Evaluated use of mobile clinics for ANC and sexually transmitted infections in India | yes |  |  |
| Kyaw, 2019[30] | 2012-2017 | Myanmar (5 clinics, 303 women) | Urban | Pregnant women living with HIV (median 29 years IQR 25–32) | Cross-sectional | Evaluate gaps and delay in ART initiation during pregnancy | yes | yes |  |
| Luseno, 2019[48] | 2014 | Kenya (9 adolescents living with HIV, 4 family members, 15 providers) | Rural | Pregnant or postpartum adolescents (age 15-19), family and providers | In-depth interviews | Examined adolescent engagement in HIV services during and after pregnancy |  |  | yes |
| Mugasha, 2014[21] | 2012 | Uganda (2 urban and 3 rural clinics, 1025 women) | Rural and urban | Pregnant women living with HIV (rural: median age 26 IQR 22-31; urban: median age 25 IQR 22-29) | Cross-sectional | Evaluated enrolment into chronic HIV care clinics for mothers and early infant diagnosis by six weeks postpartum | yes | yes | yes |
| Myer, 2015[23] | 2010-2013 | South Africa (1 clinic, 1615 women) | Urban | Pregnant women eligible for ART (age not specified) | Retrospective cohort | Compared approaches to ART initiation in pregnancy over time. | yes | yes |  |
| Price, 2014[22] | 2011-2013 | Malawi (9 clinics, 395 women) | Rural | Pregnant women living with HIV (age 15 years and older) | Retrospective cohort | Identified points of dropout on the pathway from offering HIV testing to retention on antiretroviral therapy, following the introduction of the Option B+ | yes |  | yes |
| Saleem, 2014[41] | 2012 | Uganda (11 clinics, 48 women, 11 providers) | Not specified | Nurse/midwife in charge of ANC at 11 facilities, pregnant and postpartum women living with HIV who had been referred from ANC to ART care (age range 20-42 years) | In-depth interviews | Examined barriers and facilitators of linking from ANC to long-term HIV care from patient and provider perspectives | yes |  | yes |
| Stinson, 2013[20] | 2008 | South Africa (3 clinics, 658 women) | Urban | ART eligible (CD4<200) pregnant women (median age 25 IQR 22-30) | Retrospective cohort | Assessed ART initiation in three different ANC services | yes | yes |  |
| Stover, 2019[32] | 2014-2015 | Mozambique (39 communities) | Both | All pregnant women identified in included communities (age not specified) | Case study | Describe the approach, implementation and results of community-led quality improvement to improve entry into antenatal care | yes |  | yes |
| Suryavanshi, 2018[35] | 2015-2016 | India (4 districts, 60 outreach workers) | Rural, urban | Outreach workers supporting PMTCT | Focus group discussions | Assessed challenges and opportunities with an outreach worker intervention to support PMTCT in India | yes |  | yes |
| Tsague, 2010[33] | 2006-2008 | Rwanda (32 clinics, 1622 women) | Rural, urban | Pregnant women in ANC (age not specified) | Retrospective cohort | Compared PMTCT outcomes in stand-alone sites versus integrated ART and PMTCT sites | yes | yes |  |
| Turan, 2014[46] | 2007-2009 | Kenya (8 clinics, 135 women) | Rural | Pregnant women living with HIV (aged 18 years and older) | Prospective cohort | Examined associations between linkage to HIV care, postpartum depression, and internalized stigma |  |  | yes |
| Watson-Jones, 2012[4] | 2008-2009 | Tanzania (3 clinics, 310 women) | Urban | Women newly diagnosed with HIV during pregnancy or postpartum (age range 16-48 years) as well as health providers working in PMTCT | Prospective cohort and provider interviews | Assessment of gaps when linking women from PMTCT services to ongoing HIV care. |  |  | yes |
| Weigel, 2012[40] | 2006-2009 | Malawi (1 hospital and 2 clinics, 612 women) | Urban | ART eligible pregnant women (median age 27 years IQR 24-30) | Retrospective cohort | Description of approaches used to improve ART uptake in an ART programme over time. | yes |  | yes |
| **Transition point 3 (HIV+ postpartum women transition from integrated PMTCT into non-integrated ART services soon after delivery or later postpartum): 10 studies** | | | | | | | | | |
| Buregyeya, 2017[43] | 2014 | Uganda (6 clinics, 57 women) | Rural | Pregnant and breastfeeding women living with HIV (age range 16-42 years) | In-depth interviews | Explored barriers and facilitators of the uptake and adherence to ART |  |  | yes |
| Cheshi, 2019[25] | 2014 | Nigeria (2 hospitals, 372 women) | Rural, urban | Women living with HIV who accessed PMTCT services and were referred to ART services to continue treatment postpartum (mean age 31) | Cross-sectional survey and focus group discussions | Assessed service linkage between PMTCT and adult ART services | yes | yes | yes |
| Hackett, 2019[36] | 2011-2017 | USA (1 clinic, 275 women) | Rural, urban | Pregnant women living with HIV (mean age 27 years pre- and 29 years post-implementation) | Retrospective cohort | Assessed outcomes of pregnant women living with HIV following introduction of a Perinatal Care Coordination team | yes | yes |  |
| Lubaga, 2013[45] | 2007-2010 | Uganda (1 hospital and 2 clinics, 7 stakeholders, 20 women, 112 community members) | Rural | Lost and retained women living with HIV (age range 25-48 years), their family members and health care providers | Key informant interviews, focus groups and in-depth interviews | Explored reasons for loss to follow-up |  |  | yes |
| Myer, 2017[42], and Trafford, 2018[49] | 2015-2016 | South Africa (Cohort: 1 clinic, 129 women  Qualitative: 19 women, 9 providers) | Urban | Breastfeeding postpartum women on ART (age 18 years or older) | Prospective cohort and in-depth interviews | Compared outcomes and experiences from women’s chosen postpartum ART care: Primary health care vs Adherence clubs | yes |  | yes |
| Otieno, 2010[24] | 2005 | Kenya (4 clinics, 239 women) | Urban | Mothers referred to HIV services (median age 30 IQR 23-38) | Cross-sectional | Examining the outcomes of women referred to continue HIV care after PMTCT | yes |  | yes |
| Panditrao, 2015[50] | 2002-2011 | India (10 districts, 311 women) | Rural, urban | Pregnant women living with HIV in PMTCT services (age range <20 to >40 years) | Cross-sectional | Examined factors associated with the utilization of continued care among HIV-infected women enrolled in a PMTCT program |  |  | yes |
| Phillips, 2015[13] | 2013 | South Africa (1 clinic, 279 women) | Urban | Women who started ART in pregnancy and were transferred out to general ART clinics (age 18 years or older) | Prospective cohort | Examined successful engagement in care at general ART clinics after postpartum transfer from integrated antenatal ART services | yes |  | yes |
| Phillips, 2018[26], Phillips, 2020[27] and Pellowski, 2020[47] | 2013-2018 | South Africa (Cohort: 1 clinic, 617 women  Follow-up: 450 women  Qualitative: 21 women) | Urban | Pregnant women who started ART in an integrated antenatal and ART clinic (age 18 years or older) | Additional follow up of a randomised trial cohort through routine data, a cross-section visit and in-depth interviews | Described postpartum linkage to care and retention after leaving an integrated antenatal/ART clinic after delivery. Women were randomised to transition at 6 weeks postpartum or after weaning. | yes | yes | yes |
| Winestone, 2012[44] | 2007 | Kenya (6 clinics, 36 providers) | Rural | Health providers in PMTCT services | In-depth interviews | Explored provider perspectives on integrated versus non-integrated antenatal and HIV care services. |  |  | yes |
| **Transition point 1 (HIV+ women on ART who become pregnant transition into integrated ANC/ART or non-integrated ANC and ART services) and 2 (HIV+ pregnant women not on ART transition to integrated ANC/ART or stand-alone ART and ART services to start treatment): 1 study** | | | | | | | | | |
| Geldsetzer, 2019[31] | 2012-2014 | Tanzania (36 wards) | Urban | All pregnant women aged 15 and older in the included wards | Cluster randomised control trial | To examine the impact of a community health worker intervention to improve antenatal are uptake and facility delivery. | yes |  | yes |
| **Transition points 2 (HIV+ pregnant women not on ART transition to integrated ANC/ART or stand-alone ART and ART services to start treatment) and 3 (HIV+ postpartum women transition from integrated PMTCT into non-integrated ART services soon after delivery or later postpartum): 4 studies** | | | | | | | | | |
| Anderson, 2017[38] | 2005-2013 | USA (849 births) | Urban | Women living with HIV who delivered in Philadelphia (age 16 years or older) | Retrospective cohort | Evaluating the impact of perinatal HIV case management on HIV care for pregnant and postpartum women | yes | yes |  |
| Killam, 2010[37] | 2007-2008 | Zambia (8 clinics, 1566 women) | Urban | Pregnant women eligible for ART (mean age 27 years) | Stepped-wedge trial | Evaluated integrated antenatal/ART care and referral from ANC to ART services | yes |  |  |
| van Lettow, 2014[15] | 2012-2013 | Malawi (114 clinics) | Rural, urban | Women with HIV presenting for ANC (age not reported) | Retrospective cohort | Evaluating ART uptake and retention under different models of care. |  | yes |  |
| White, 2013[53] | 2010 | Cambodia (1 district, 82 women) | Urban and rural | Pregnant women living with HIV who delivered after the implementation of a “linked response” intervention (age not reported) and health providers | In-depth interviews and focus groups | Assessed provider and user perspectives on a pilot integrated reproductive health service. |  |  | yes |
| **All points of transition: 1 study** | | | | | | | | | |
| Besada, 2018[39] | 2015 | Cote D’Ivoire, DRC, Malawi, Uganda | Not specified | Key informants in five countries on the role of community cadres in PMTCT | In-depth interviews and focus groups | Explored the role of community cadres in improving access and retention in PMTCT | yes |  |  |

ANC – antenatal care; ART – antiretroviral therapy; DRC – Democratic Republic of Congo; PMTCT – prevention of mother-to-child HIV transmission
